# Myeloid-derived interleukin-10 induced by thrombospondin-1 mediates host defense and regulates inflammation during acute bacterial lung infection

**DOI:** 10.1128/iai.00614-25

**Published:** 2026-02-24

**Authors:** Agnes Jara-Collao, Francisca Román, Gonzalo Torres-Ayala, Felipe Novoa, Cynthia Moreno-Quidecoy, Aillairlyn Jara, Pablo A. González, Susan M. Bueno, Alexis M. Kalergis, Hernán F. Peñaloza

**Affiliations:** 1 Millennium Institute on Immunology and Immunotherapy, Facultad de Ciencias Biológicas, Pontificia Universidad Católica de Chile 98732 , Santiago, Chile; 2 Departamento de Endocrinología, Facultad de Medicina, Escuela de Medicina, Pontificia Universidad Católica de Chile 60709 , Santiago, Chile; 3 Departamento de Laboratorios Clínicos, Escuela de Medicina, Facultad de Medicina, Pontificia Universidad Católica de Chile 60709 , Santiago, Chile; Stanford University School of Medicine, Stanford, California, USA

**Keywords:** thrombospondin-1, interleukin-10, neutrophils, *Pseudomonas aeruginosa*

## Abstract

*Pseudomonas aeruginosa* (*P. aeruginosa*) is a leading cause of hospital- and ventilator-associated pneumonia, driven by virulence factors that damage lung tissue. Neutrophils and monocytes are myeloid immune cells that play key roles in host defense during pulmonary infection against *P. aeruginosa*; although if not properly regulated, their inflammatory activity can lead to severe lung injury. Thrombospondin-1 (TSP-1) is a host glycoprotein that regulates neutrophil activation in the lungs during *P. aeruginosa* infection, protecting the host from mortality. Here, we demonstrate that in addition to regulating the pro-inflammatory phenotype of neutrophils, during *P. aeruginosa* infection, TSP-1 induces IL-10 in the lung tissue of mice inoculated with this bacterium. Our data show that neutrophils and Ly6C^+^ monocytes are major sources of lung IL-10 during the first 48 h post-infection, a cytokine that is required for host survival, host defense, and to reduce lung inflammation and injury. Further *in vitro* studies determined that TSP-1 induces IL-10 production in lipopolysaccharide-stimulated CD11b^+^Ly6G^+^Ly6C^+^ cells differentiated from bone marrow precursors through a mechanism dependent on the transcription factor peroxisome proliferator-activated receptor gamma (PPARγ). Importantly, intratracheal transfer of *IL-10^+/+^* CD11b^+^Ly6G^+^Ly6C^+^ cells 1 day before infection to *Thbs1^−/−^* mice restored their ability to produce IL-10 in the lungs, improved host defense, and reduced lung inflammation and permeability upon infection. Altogether, our data show that TSP-1 induces IL-10 production in neutrophils and monocytes, enhancing host defense while reducing lung inflammation.

## INTRODUCTION

Pneumonia is the leading cause of death among children under five and the elderly worldwide (1). Defined as an inflammation and injury of the alveolar space, hospital-acquired pneumonia and ventilator-associated pneumonia, mostly caused by opportunistic antibiotic-resistant bacteria such as *Pseudomonas aeruginosa* (*P. aeruginosa*), are associated with high mortality rates ranging from 20% to 50% (2, 3). *P. aeruginosa* mainly infects patients admitted to the Intensive Care Unit (ICU) (2, 4) and often leads to severe concomitant diseases such as Acute Respiratory Distress Syndrome (ARDS), which involves acute respiratory failure characterized by diffuse lung edema of the alveolar-capillary barrier (5). When *P. aeruginosa* reaches the alveolar space, bacterial virulence factors, including ExoTUYS toxins or elastase (LasB), are injected into host cells and secreted to the alveolar space, respectively, causing epithelial and endothelial cell death and lung injury (6–8). In response, bacterial recognition by epithelial cells and alveolar macrophages elicits the production of antimicrobial peptides, cytokines, and chemokines that recruit neutrophils and monocytes to eliminate the bacteria (9, 10).

Neutrophils and monocytes are essential immune effector cells against *P. aeruginosa* (9). Neutrophils eliminate bacteria during infection through several mechanisms. These include phagocytosis, degranulation, and neutrophil extracellular trap (NET) release (11), biological processes that if not properly regulated, can trigger inflammation and cause injury (12, 13). Therefore, neutrophil function during *P. aeruginosa* lung infection must be tightly regulated. It is now well established that both human and mouse neutrophils are plastic cells that can exhibit either pro- or anti-inflammatory phenotypes during infection (14). Anti-inflammatory neutrophils suppress the pro-inflammatory activity of various immune cells through multiple mechanisms, including the production of interleukin-10 (IL-10) (14–16). The presence of IL-10-producing neutrophils has been identified in the lungs of mice during bacterial infection caused by *Klebsiella pneumoniae* (16) and *Streptococcus pneumoniae* (17)**,** with bacterial Pathogen-Associated Molecular Patterns (PAMPs) triggering IL-10 in these cells through their recognition by Pattern Recognition Receptors (PRRs) (18). In humans, identifying these cells was more challenging, yet the integration of transcriptomic, proteomic, and flow cytometry data during the COVID-19 pandemic showed that the functional and phenotypic heterogeneity of neutrophils observed in mice is also present in humans (19, 20).

Although various host factors, including type I interferon, Prostaglandin E2, and vitamin D, can induce IL-10 production (21), the nature of host-derived molecules that stimulate IL-10 production in myeloid cells during infection and the mechanisms involved remain unclear.

Thrombospondin-1 (TSP-1) is a host matricellular glycoprotein that interacts with different types of ligands, including surface receptors involved in immunity (CD36, CD148, and CD47) and bacterial/neutrophil-derived proteases (22). Previous studies show that TSP-1 plays a central protective and immunomodulatory role in the lung immune response during acute *P. aeruginosa* infection (23, 24). Notably, these studies have shown that a Kazal-like sequence (DNCQYVYNV) in the type 3 repeat domain of TSP-1 inhibits the proteolytic activity of both neutrophil-derived proteases (neutrophil elastase, cathepsin G) and *P. aeruginosa*-derived elastase LasB, *in vivo* and *in vitro* (23–25). During acute *P. aeruginosa* pneumonia in mice, TSP-1 regulates neutrophil recruitment and activation, facilitating an adequate bacterial clearance, limiting lung injury, and increasing host survival (23, 24). Mechanistically, *in vivo* data have shown that TSP-1 regulates lung neutrophil recruitment and activation by downregulating the upstream factor IL-36γ (23). IL-36γ is a pro-inflammatory cytokine that, once released into the extracellular space, is activated by host and microbial proteases that cleave IL-36γ at Y^16^, S^18^, or M^19^ (23). In the airspace, the cleaved or activated forms of IL-36γ induce the production of CXCL-1, CXCL-2, GM-CSF, and IL-1β, which promote neutrophil recruitment and activation, as evidenced by the release of NE and myeloperoxidase (MPO) (23).

Additionally, TSP-1 stimulates IL-10 production by lung macrophages during the resolution phase in an experimental lipopolysaccharide (LPS)-induced lung injury model (26). Given that TSP-1 regulates pro-inflammatory neutrophil activity and induces IL-10 production in macrophages, we hypothesized that TSP-1 promotes IL-10 production by lung neutrophils recruited during acute *P. aeruginosa* lung infection. Our data show that *in vivo*, TSP-1 induces IL-10 production in the lungs during acute *P. aeruginosa* infection, with neutrophils and monocytes being highly heterogeneous cells and the primary myeloid sources of IL-10 during the first 48 h post-infection. Additionally, *in vitro* studies suggest that TSP-1 induces IL-10 production in these cells through the activity of the transcription factor PPARγ, and its production is necessary for host defense and regulation of lung inflammation during acute infection of TSP-1-deficient mice.

## RESULTS

### Thrombospondin-1 is necessary for lung IL-10 production and effective immunity against *P. aeruginosa*

**Fig 1:**
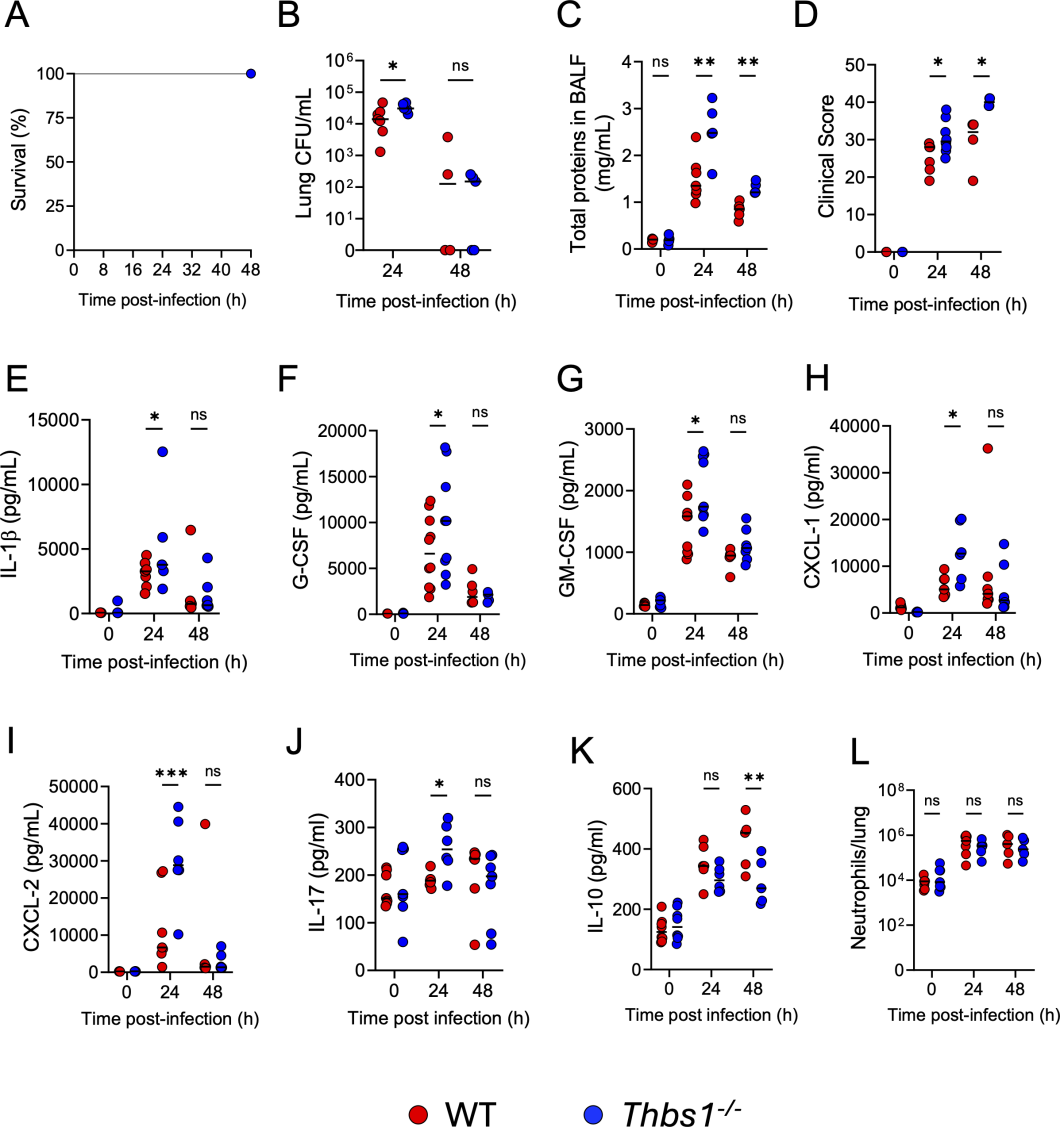
Thrombospondin-1 improves host defense, protects from lung micro-permeability, and induces lung IL-10 production during *P. aeruginosa* infection. WT and thrombospondin-1-deficient (*Thbs1^−/−^*) mice were intratracheally inoculated with 1 × 10^6^ CFU of *P. aeruginosa*. (**A**) Survival was evaluated during the first 48 h post-infection (hpi), (**B**) lung bacterial burden (CFU/mL), (**C**) total protein content in the bronchoalveolar lavage fluid (BALF), (**D**) disease severity and lung cytokine levels of (**E**) IL-1β, (**F**) G-CSF, (**G**) GM-CSF, (**H**) CXCL-1, (**I**) CXCL-2, (**J**) IL-17, (**K**) IL-10, and (**L**) lung neutrophil numbers were evaluated at 0, 24, and 48 h post-infection. Two-tailed *t*-test was used for single comparisons between WT (0 hpi, *n* = 5–8; 24 hpi *n* = 6–10; 48 hpi, *n* = 4–10) and *Thbs1^−/−^* (0 hpi, *n* = 5–8; 24 hpi *n* = 5–9; 48 hpi, *n* = 4–7) mice (ns *P* > 0.05, *P < 0.05, **P < 0.01, ***P < 0.001). Each data point represents an individual mouse, combined from two independent experiments. Lines indicate the median.

In our experimental setting, we show that wild type (WT) and TSP1-deficient (*Thbs1^−/−^*) mice intratracheally inoculated with *P. aeruginosa* (PA14, 1 × 10^6^ CFU) present a 100% survival during the first 48 h post-infection (hpi) (Fig. 1A). Despite no differences in survival and consistent with previous studies (23, 24), our data demonstrate that *Thbs1^−/−^* mice exhibit impaired lung bacterial clearance at 24 h compared with WT mice (Fig. 1B). The impaired host defense observed at 24 hpi is linked to increased lung micro-permeability (Fig. 1C), heightened disease severity (Fig. 1D), and elevated levels of IL-1β, G-CSF, GM-CSF, CXCL-1, CXCL-2, and IL-17 in the lungs (Fig. 1E through J). Although no significant differences were seen regarding lung bacterial burden (Fig. 1B) or pro-inflammatory cytokine/chemokine production between *Thbs1^−/−^* and WT mice at 48 hpi (Fig. 1E through J), *Thbs1^−/−^* mice showed increased lung micro-permeability (Fig. 1C), greater disease severity (Fig. 1D), and reduced production of the anti-inflammatory cytokine IL-10 (Fig. 1K), compared to WT mice at this time point. The increased lung micro-permeability and disease severity observed in *Thbs1^−/−^* mice is not directly related to differences in lung neutrophils, as equivalent numbers of neutrophils were observed in the lungs of WT and *Thbs1^−/−^* mice at 24 and 48 hpi (Fig. 1L), suggesting that these differences between WT and Thbs1^−/−^ are due to potential defective neutrophil function.

### Lung IL-10 is required for host defense and survival during *P. aeruginosa* infection

**Fig 2:**
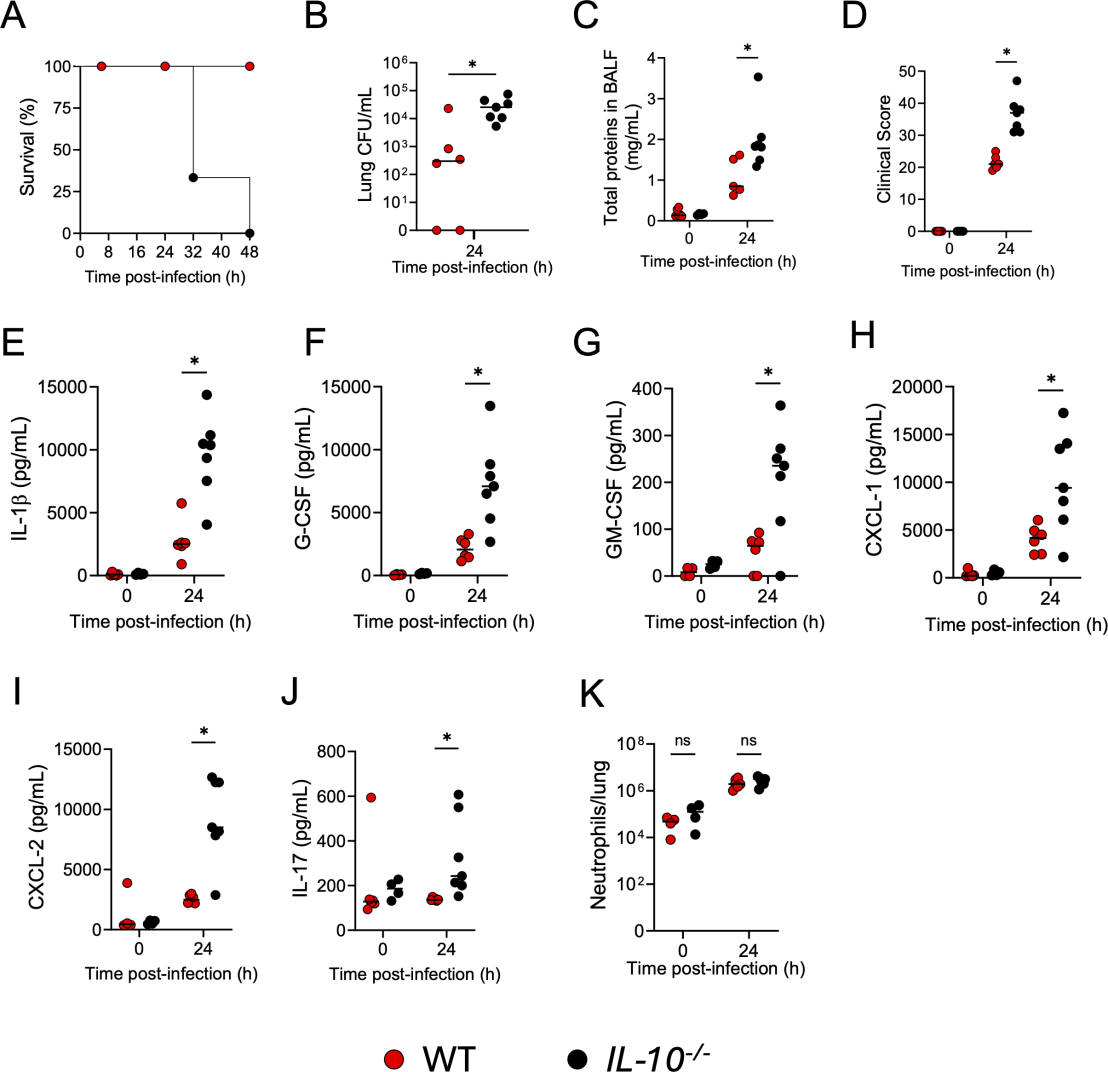
IL-10 mediates host survival, host defense, and lung cytokines during *P. aeruginosa* infection. WT and IL-10-deficient (*IL-10^−/−^*) mice were intratracheally inoculated with 0.5 × 10^6^ CFU of *P. aeruginosa*. (**A**) Survival was evaluated during the first 48 h post-infection (hpi), (**B**) lung bacterial burden (CFU/mL), (**C**) total BALF protein content, (**D**) clinical score, lung pro-inflammatory cytokine production (**E**) IL-1β, (**F**) G-CSF, (**G**) GM-CSF, (**H**) CXCL-1, (**I**) CXCL-2, (**J**) IL-17, and (**K**) lung neutrophil numbers were measured at 0 and 24 hpi. A two-tailed *t*-test was used for single comparisons between WT (0 hpi *n* = 4–6; 24 hpi *n* = 6) and *IL-10^−/−^* (0 hpi *n* = 4; 24 hpi *n* = 7) mice (ns *P* > 0.05, *P < 0.05). Each data point represents an individual mouse. Lines indicate the median.

IL-10 is an anti-inflammatory cytokine that plays a key role in the host immune response to various pulmonary bacterial infections, including *Streptococcus pneumoniae* and *Klebsiella pneumoniae* (14, 27). Since TSP-1 deficiency resulted in lower lung IL-10 levels during infection, impaired immune defense, and increased inflammation, we examined the role of IL-10 in host defense and inflammation during acute *P. aeruginosa* lung infection using IL-10-deficient mice (*IL-10^−/−^*). Our data show that *IL-10^−/−^* mice inoculated with 1 × 10^6^ CFU showed a 75% of mortality during the first 24 hpi, while WT mice showed 0% mortality (Fig. S1). To avoid survival bias, WT and *IL-10^−/−^* mice were inoculated with a lower dose of 0.5 × 10^6^ CFU of PA14, showing in both cases 0% mortality at 24 hpi; although at 48 hpi, *IL-10^−/−^* mice showed a 100% mortality, while WT mice registered 0% mortality (Fig. 2A). In addition, *IL-10^−/−^* mice inoculated with 0.5 × 10^6^ CFU of PA14 exhibited an increased lung bacterial burden (Fig. 2B), elevated lung micro-permeability (Fig. 2C), and higher disease severity (Fig. 2D) at 24 hpi compared with WT mice.

The heightened lung cytotoxicity, micro-permeability, and disease severity observed in *IL-10^−/−^* mice at 24 hpi aligned with increased production of IL-1β, G-CSF, GM-CSF, CXCL-1, CXCL-2, and IL-17 in their lungs compared to WT mice (Fig. 2E through J) although equivalent numbers of neutrophils were found in the lungs of *IL-10^−/−^* and WT mice at 24 hpi (Fig. 2K). These data indicate that during acute pulmonary *P. aeruginosa* infection, IL-10 production within the first 48 h is essential to downregulate the lung inflammatory response, promote host defense, and reduce lung micro-permeability, ultimately being necessary for host survival.

### Neutrophils and Ly6C^+^ monocytes are the primary sources of lung IL-10 during acute *P. aeruginosa* infection

**Fig 3:**
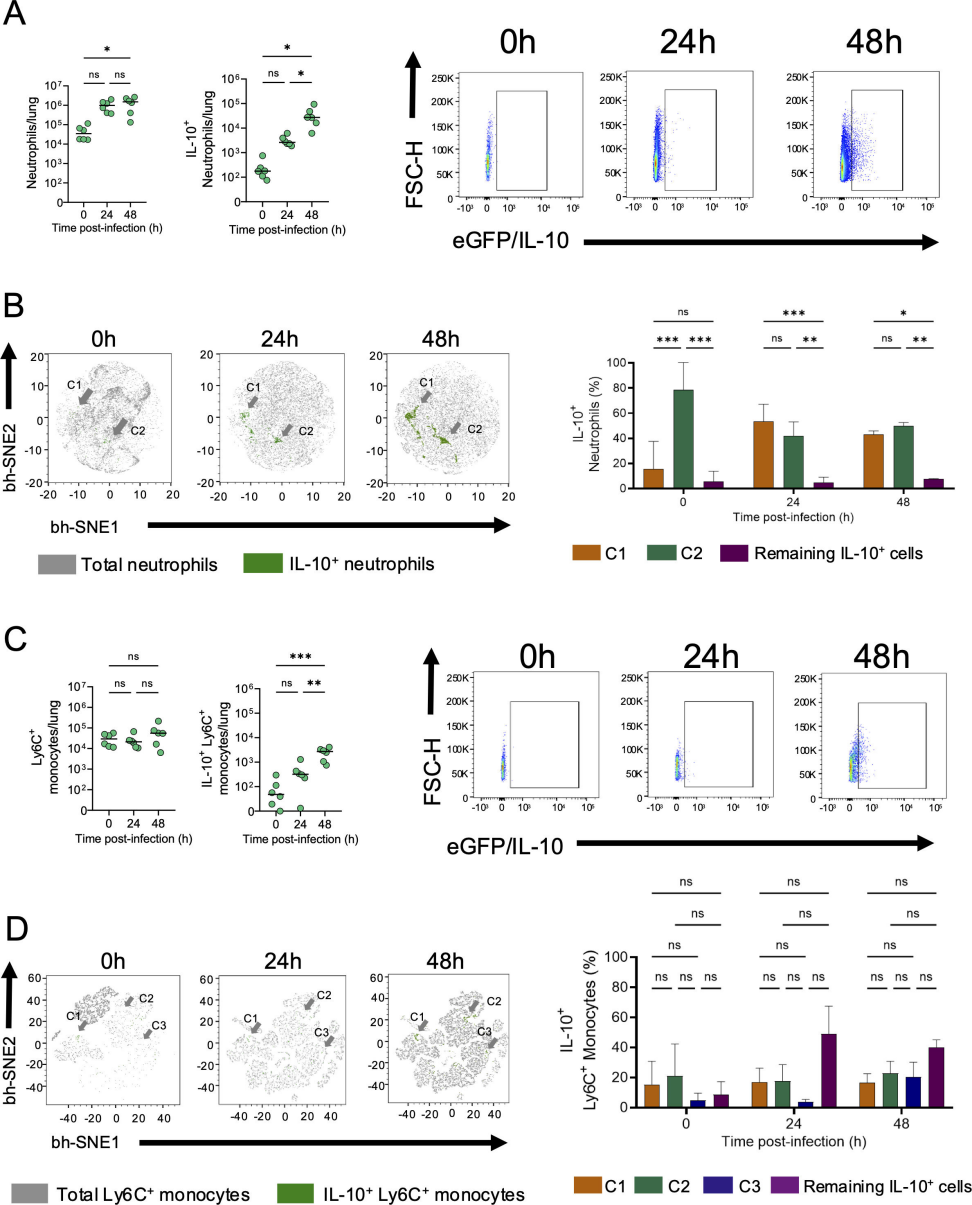
Neutrophils and Ly6C^+^ monocytes are the main sources of lung IL-10 in response to *P. aeruginosa* infection. IL-10-eGFP mice (0 hpi, *n* = 6; 24 hpi, *n* = 6; 48 hpi, *n* = 5–6) were intratracheally inoculated with 1 × 10^6^ CFU of *P. aeruginosa,* and IL-10 production by myeloid cells was evaluated by traditional and multiparametric flow cytometry at 0, 24, and 48 hpi (see Fig. S2 for gating strategy). Total lung cells, IL-10^+^ cells, representative dot-plots and are shown for (**A**) neutrophils, along with (**B**) bh-SNE-plots depicting total and IL-10^+^ neutrophils grouped in clusters and their respective quantification. In parallel, (**C**) lung IL-10-producing Ly6C^+^ monocytes were quantified by flow cytometry and by (**D**) bh-SNE-plots depicting total and grouped/scattered IL-10^+^ monocytes with their respective quantification. Gray arrows indicate clustered IL-10^+^ cells. An ordinary one-way analysis of variance (ANOVA) test was performed followed by a Holm Sidak post-hoc for multiple comparisons over time (ns *P* > 0.05, *P < 0.05, **P < 0.01, ***P < 0.001). Each data point represents an individual mouse, combined from two independent experiments. Lines indicate the median.

Next, we used a reporter mouse that encodes enhanced (e)GFP along with IL-10 (28) to evaluate the cellular source of IL-10 in the lungs of *P. aeruginosa*-inoculated mice during the first 48 hpi. This mouse line has been previously validated to measure IL-10 production by lung immune cells during bacterial infection (14, 17). Intratracheal inoculation of IL-10-eGFP mice with *P. aeruginosa* showed 100% survival (Fig. S2A) and a reduction in lung bacterial burden at 24 hpi compared to the initial inoculum that remained stable at 48 hpi (Fig. S2B), along with a swift and sustained increase in disease severity (Fig. S2C)**,** and elevated lung micro-permeability at 24 hpi that rapidly returned to baseline levels at 48 hpi (Fig. S2D). Additionally, these mice exhibited a transient increase in IL-1β, G-CSF, GM-CSF, CXCL-1, and CXCL-2 at 24 hpi, while IL-17 remained unchanged (Fig. S2E through J), and a quick production of IL-10 at 24 and 48 hpi (Fig. S2K). We further identified the production of lung IL-10 by different lung-recruited immune cells using flow cytometry (Fig. S3), as previously described (16, 17). Our data show that neutrophils (CD11b^+^Ly6G^+^Ly6C^+^) are rapidly recruited during the first 24 hpi and produce high levels of IL-10 at 48 hpi, making them the most abundant IL-10-producing cells among the identified populations at this time point (Fig. 3A).

Neutrophils are well-recognized heterogeneous cells with respect to transcriptomic signatures, as well as in terms of phenotypic and functional profiles in health and disease (17, 29–32). Given that heterogeneity, through a Barnes-Hut Stochastic Neighbor Embedding (bh-SNE) analysis, we visualized the spatial distribution of IL-10^+^ neutrophils in the lungs during the first 48 h post *P. aeruginosa* infection. Importantly, the percentage of IL-10^+^ neutrophils relative to total neutrophils at each time point, using this methodology, is comparable to that obtained with traditional flow cytometry (Fig. S4). This analysis also demonstrated that the IL-10^+^ neutrophil population groups in at least two different clusters that are significantly enriched at 48 hpi, comprising 90% of total IL-10^+^ neutrophils and highlighting their heterogeneity (Fig. 3B). The second most abundant myeloid source of IL-10 identified in the lungs during *P. aeruginosa* infection was classical Ly6C^+^ monocytes (CD11b^+^Ly6G^neg^Ly6C^+^), cells that also showed significant IL-10 production at 48 hpi (Fig. 3C). Bh-SNE analyses of Ly6C^+^ monocytes revealed major differences in their distribution before and after infection (Fig. 3D). In addition, unlike IL-10^+^ neutrophils, IL-10^+^ monocytes showed a much more scattered distribution among the total population, identifying three small clusters that, together, comprise around 60% of IL-10^+^ monocytes, while the remaining 40% were scattered cells (Fig. 3D).

No significant IL-10 production was observed from alveolar macrophages, B and T cells, eosinophils, CD11b^+^ and CD11b^neg^ dendritic cells, alternative Ly6C^neg^ monocytes, interstitial macrophages, or monocyte-derived macrophages at 24 or 48 hpi (Fig. S5).

### TSP-1 controls IL-10 production by myeloid cells *in vitro*

**Fig 4:**
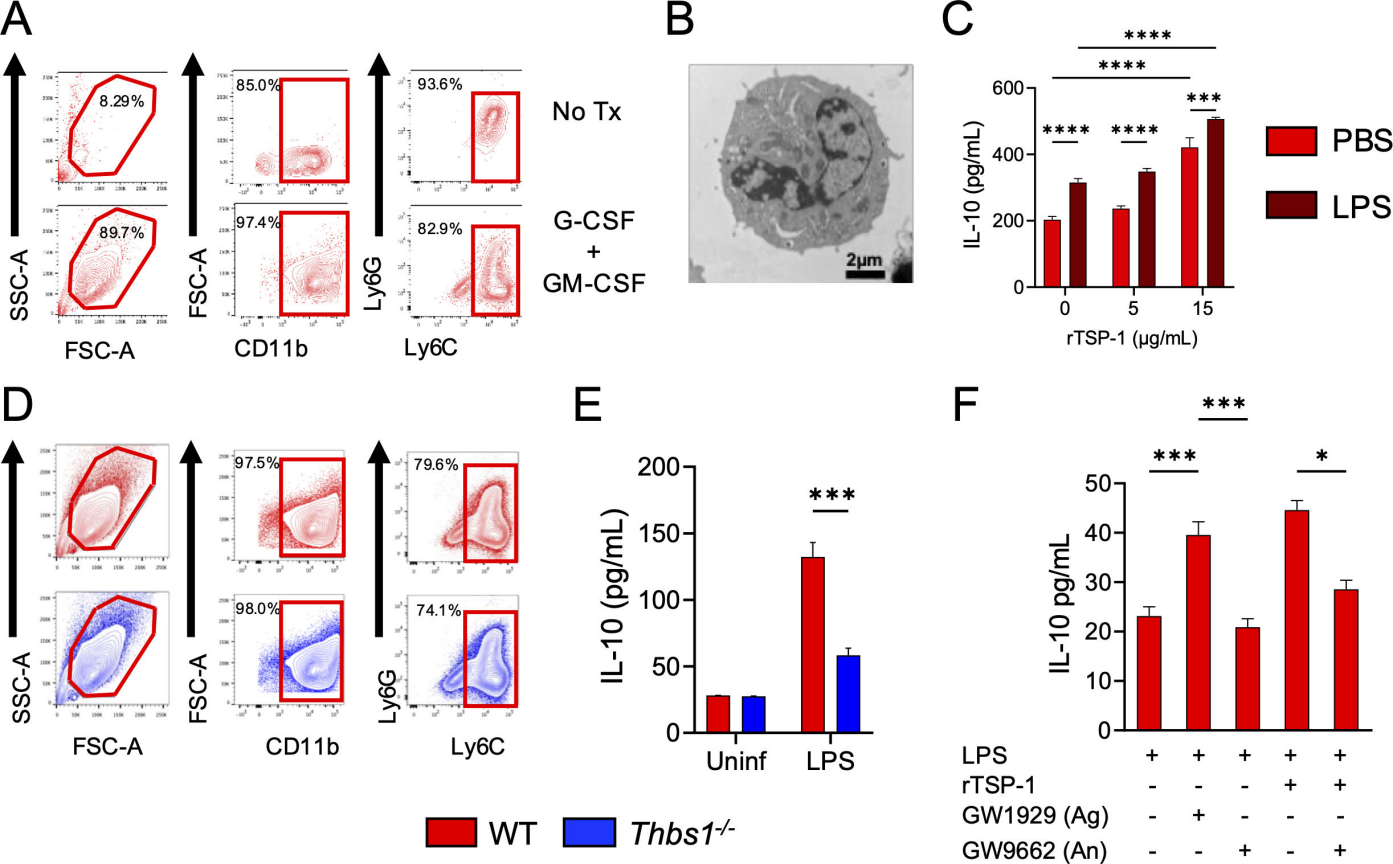
TSP-1 induces IL-10 production by CD11b^+^Ly6G^+^Ly6C^+^ cells *in vitro*. Mouse bone marrow-precursors were incubated in complete RPMI medium with or without G-CSF/GM-CSF (40 ng/mL) for 5 days to obtain CD11b^+^Ly6G^+^Ly6C^+^ cells. (**A**) Phenotypic characterization of differentiated cells by flow cytometry and by (**B**) transmission electron microscopy. (**C**) IL-10 production in the supernatant of CD11b^+^Ly6G^+^Ly6C^+^ cells incubated with rTSP-1 (0, 5, or 15 µg/mL) for 24 h and stimulated with LPS (100 ng/mL) or vehicle (PBS) for 4 h. (**D**) Phenotypic characterization of WT and *Thbs1^−/−^* CD11b^+^Ly6G^+^Ly6C^+^ cells by flow cytometry. (**E**) IL-10 production by WT or *Thbs1^−/−^* CD11b^+^Ly6G^+^Ly6C^+^ cells stimulated with LPS (100 ng/mL) or vehicle (PBS). (**F**) CD11b^+^Ly6G^+^Ly6C^+^ cells were pre-incubated with rTSP-1 (15 µg/mL) for 24 h and with the PPARγ agonist (GW1929, 100 µM), antagonist (GW9662,10 µM), or vehicle (DMSO) for 1 h. Cells were stimulated with LPS (100 ng/mL) for 4 h, and IL-10 production was assessed by ELISA in the supernatant. (**C**) Two-way ANOVA following Holm Sidak post-hoc test was used for IL-10 production in CD11b^+^Ly6G^+^Ly6C^+^ cells pre-incubated with rTSP-1. (**E**) Two-tailed *t*-test was used to evaluate the production of IL-10 between WT and *Thbs1^−/−^* CD11b^+^Ly6G^+^Ly6C^+^ cells stimulated with bacterial LPS (***P < 0.001, ****P < 0.0001). (**F**) One-way ANOVA following Holm Sidak post-hoc test was used for IL-10 production by CD11b^+^Ly6G^+^Ly6C^+^ cells pre-treated with PPARγ agonist and antagonist (*P < 0.05, ***P < 0.001).

Given that neutrophils and Ly6C^+^ monocytes were the primary identified cellular sources of IL-10 in the lung tissue during the first 48 h after *P. aeruginosa* infection, and due to short lifespan of bone-marrow isolated neutrophil in culture (Fig. S6), we differentiated CD11b^+^Ly6G^+^Ly6C^+^ cells from bone marrow precursors (16, 33) (Fig. 4A) to study *in vitro* the ability of TSP-1 to induce IL-10 in these cells. Differentiated CD11b^+^Ly6G^+^Ly6C^+^ cells not only expressed surface markers similar to those of lung neutrophils and monocytes but, like neutrophils, were identified as polymorphonuclear cells by transmission electron microscopy (Fig. 4B). When these cells were incubated with recombinant TSP-1 (rTSP-1; 0, 5, and 15 µg/mL), high levels of IL-10 were produced even without stimulation with LPS *in vitro* (Fig. 4C), suggesting that TSP-1 can induce by itself the production of IL-10 in these cells. Still, it can also synergize with the intrinsic ability of LPS to induce IL-10 production, as cells incubated with rTSP-1 (15 µg/mL) and stimulated with LPS produced higher levels of IL-10 compared to cells treated with rTSP-1 or LPS alone (Fig. 4C). Similarly, CD11b^+^Ly6G^+^Ly6C^+^ cells differentiated from *Thbs1^−/−^* mice (Fig. 4D) produced less IL-10 after LPS stimulation than WT cells (Fig. 4E). Previous studies have shown that PPARγ is an important mediator of IL-10 production in myeloid cells during bacterial lung infection (34). Therefore, we evaluated in our model whether the induction of IL-10 by TSP-1 in myeloid cells depends on PPARγ. *In vitro*, differentiated CD11b^+^Ly6G^+^Ly6C^+^ cells stimulated with LPS exhibited increased IL-10 production in the presence of the PPARγ agonist GW1929 compared to vehicle-treated cells, but not in the presence of the antagonist GW9662 when cells were stimulated with LPS (Fig. 4F). These data suggest that PPARγ mediates IL-10 production in response to LPS. Additionally, TSP-1-mediated IL-10 induction in LPS-stimulated cells was blocked when cells were incubated with the antagonist GW9662 (Fig. 4F), suggesting that, at least *in vitro*, TSP-1 induces IL-10 in myeloid cells through a mechanism dependent on PPARγ.

### IL-10 production by CD11b^+^Ly6G^+^Ly6C^+^ cells enhances host defense and decreases lung micro-permeability in TSP-1-deficient mice

**Fig 5:**
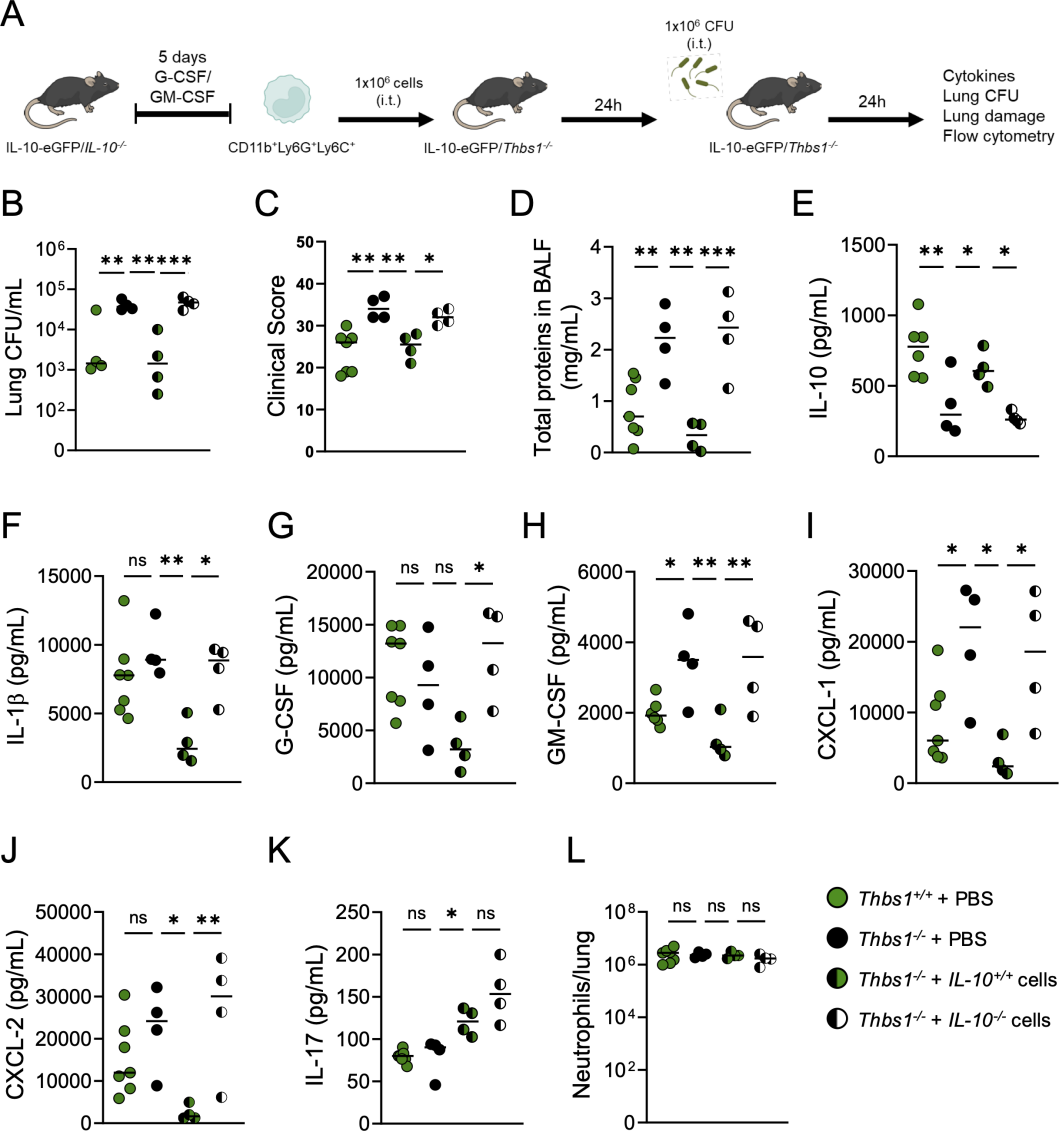
IL-10 production by CD11b^+^Ly6G^+^Ly6C^+^ cells improves host defense and reduces lung micro-permeability in TSP-1-deficient mice. (**A**) *IL-10^+/+^* CD11b^+^Ly6G^+^Ly6C^+^ cells (from IL-10-eGFP mice) and *IL-10^−/−^* CD11b^+^Ly6G^+^Ly6C^+^ cells (from *IL-10^−/−^* mice) were differentiated and intratracheally transferred to *Thbs1^−/−^* mice (non-transferred IL-10-eGFP mice [*Thbs1^+/+^*] and non-transferred *Thbs1^−/−^* mice were used as controls). Twenty-four hours after transfer, mice were intratracheally inoculated with 1 × 10^6^ CFU of PA14 and (**B**) lung bacterial burden, (**C**) clinical score, (**D**) total BALF protein content were measured, and (**E**) IL-10, (**F**) IL-1β, (**G**) G-CSF, (**H**) GM-CSF, (**I**) CXCL-1, (**J**) CXCL-2, (**K**) IL-17, and (**L**) lung neutrophils were measured in lung homogenates at 24 h post-infection. One-way ANOVA following Holm-Sidak post-hoc test was used for lung bacterial burden, clinical score, total BALF protein content, and cytokine production (ns *P* > 0.05, *P < 0.05, **P < 0.01, ***P < 0.001). Each data point represents an individual mouse combined from two independent experiments. Lines indicate the median.

Finally, we hypothesized that the lack of IL-10 production by myeloid polymorphonuclear cells due to TSP-1 deficiency is a key determinant of the impaired host defense and the dysregulated lung inflammation observed in *Thbs1^−/−^* mice during acute *P. aeruginosa* infection. Given that CD11b^+^Ly6G^+^Ly6C^+^ cells differentiated from WT mice produce IL-10 in response to *P. aeruginosa*, while those from IL-10-deficient mice cannot (*IL-10^−/−^*) (Fig. S7), to address this hypothesis, we differentiated these cells from IL-10-eGFP (*IL-10^+/+^*) and from IL-10 deficient (*IL-10^−/−^*) mice and intratracheally transferred to *Thbs1^−/−^* mice. 24 hours after cell transfer, mice were inoculated with *P. aeruginosa,* and lung bacterial burden, lung micro-permeability, cytokine production, and neutrophil recruitment were evaluated at 24 h post-infection (Fig. 5A). Transfer of *IL-10^+/+^* CD11b^+^Ly6G^+^Ly6C^+^ cells to *Thbs1^−/−^* mice significantly reduced lung bacterial burden compared to *Thbs1^−/−^* mice transferred with *IL-10^−/−^* cells and those untransferred (PBS) (Fig. 5B). Consistently, *Thbs1^−/−^* mice transferred with *IL-10^+/+^* CD11b^+^Ly6G^+^Ly6C^+^ cells showed reduced disease severity (Fig. 5C) and decreased lung micro-permeability (Fig. 5D) compared to untransferred *Thbs1^−/−^* mice. Surprisingly, the transfer of *IL-10^+/+^* CD11b^+^Ly6G^+^Ly6C^+^ cells alone restored global lung IL-10 production of *Thbs1^−/−^* mice (Fig. 5E), produced, in part, by transferred cells identified by flow cytometry in the lungs of transferred mice based on eGFP expression (Fig. S8), and reduced the production of key cytokines such as IL-1β, G-CSF, GM-CSF, CXCL-1, and CXCL-2 although it increased IL-17 production (Fig. 5F through K). Finally, the transfer of CD11b^+^Ly6G^+^Ly6C^+^ cells did not affect the recruitment of total neutrophils in the lungs during infection (Fig. 5F through L).

These findings indicate that IL-10 production by myeloid polymorphonuclear cells is essential for effective host defense against acute pulmonary *P. aeruginosa* infection in mice. They also strongly suggest that the impaired host defense and heightened inflammation observed in *Thbs1^−/−^* mice are partly due to a deficiency in IL-10 production during the first 48 hpi.

## DISCUSSION

Previous studies have shown that TSP-1 prevents neutrophil hyperactivation and recruitment to the lungs during acute *P. aeruginosa* infection (23). In the present study, we present evidence indicating that during acute lung *P. aeruginosa* infection, TSP-1 stimulates neutrophils (CD11b^+^Ly6G^+^Ly6C^+^) to produce IL-10 within the first 48 h of infection. IL-10 serves as a key immunoregulatory cytokine during acute bacterial pulmonary infection. During infection caused by the Gram-positive pathogen *Streptococcus pneumoniae*, early IL-10 production in the lungs—mainly by neutrophils—reduced lung inflammation and neutrophil recruitment while also improving mice survival (17, 27). Additionally, during carbapenem-resistant *Klebsiella pneumoniae* pulmonary infection, IL-10 produced by neutrophils and monocytic-myeloid-derived suppressor cells prevented lung injury, regulated cytokine production, aided lung bacterial clearance, and improved mouse survival (16). In the context of pulmonary *P. aeruginosa* infection, IL-10 production is beneficial for the host as it enhances lung bacterial clearance and protects against lung injury and elevated IL-6 expression at 10 hpi in mice (35). Additionally, a model of chronic pulmonary *P. aeruginosa* infection using agarose beads embedded with *P. aeruginosa* showed that instillation of recombinant IL-10 improved the survival of infected mice and decreased lung inflammation, compared to the placebo control group, indicating that IL-10 protects the host during pulmonary *P. aeruginosa* infection (36). While our data align with these findings, we also offer new insights into the role of IL-10 in early survival against *P. aeruginosa,* particularly highlighting the IL-10 involvement in regulating the production of multiple cytokines and chemokines involved in neutrophil recruitment and activation. Additionally, we have identified neutrophils and Ly6C^+^ monocytes as active sources of lung IL-10 during the first 48 h after *P. aeruginosa* infection.

Considering the pro- and anti-inflammatory diversity of neutrophils and monocytes during lung infection, we have used an *in vitro* model of differentiated polymorphonuclear CD11b^+^Ly6G^+^Ly6C^+^ cells biased toward an anti-inflammatory phenotype (37). These cells have previously been phenotypically and functionally characterized by flow cytometry, and it is well known that they express the classical neutrophil/monocyte surface markers CD11b, Ly6C, and Ly6G, suppress T cell cytotoxicity *in vitro*, and produce IL-10 in response to inflammatory stimuli (16, 33, 37). Using transmission electron microscopy, we showed that these cells have a polymorphonuclear phenotype, providing novel insights regarding their biology. Given that bone marrow-isolated neutrophils offer several intrinsic challenges for *in vitro* studies, including short life-span, pro-inflammatory bias, and rapid degranulation/NETosis in response to microbes in culture (33, 38), the longer lifespan, anti-inflammatory bias, neutrophil surface marker expression, and polymorphonuclear phenotype of CD11b^+^Ly6G^+^Ly6C^+^ cells make them a relevant model to study whether TSP-1 can directly induce IL-10 production in neutrophils and to identify the underlying mechanisms.

These assays demonstrated that *in vitro*, differentiated CD11b^+^Ly6G^+^Ly6C^+^ cells stimulated with rTSP-1 produced IL-10 even in the absence of any inflammatory stimulus. These data align with previous studies showing that distinct host factors, including vitamin D3, type-I interferon, and prostaglandin E2, induce IL-10 production across different inflammatory contexts (21, 39). Additionally, the production of IL-10 in TSP-1-incubated cells was even more accentuated when cells were stimulated with LPS. It is well-known that several PAMPs including LPS can induce IL-10 production after they are recognized by PPRs (21). We also demonstrate that live *P. aeruginosa* can also induce high levels of IL-10 in these cells, presumably through LPS and flagellin, virulence factors that induce IL-10 through the recognition and intracellular pathways mediated by the toll-like receptors 4 and 5, respectively (40, 41).

PPARγ is a major regulator of IL-10 production by myeloid cells in various inflammatory contexts, including dextran sodium sulfate-induced colitis in rats (42), Parkinson’s disease in mice (43), and lung bacterial infection in mice (34) due to the presence of PPAR/RXR response elements (PPAR-RE) in the promoter region of the *il10* gene (44–46). In the context of acute pulmonary infection caused by *K. pneumoniae* in mice, PPARγ regulates IL-10 production in lung myeloid-derived suppressor cells (CD11b^+^F4/80^+^Ly6C^+^Ly6G^int^), as evidenced by a reduction in IL-10 production when cells were *ex vivo* incubated with the PPARγ antagonist GW9662 (34).

Our data suggest that TSP-1 stimulates IL-10 production in CD11b^+^Ly6G^+^Ly6C^+^ cells via PPARγ *in vitro*. Due to its structural properties, TSP-1 binds to multiple ligands involved in various cellular processes, such as matrix organization, cell-cell adhesion, coagulation and platelet activation, angiogenesis, tissue development, and immune responses (47). Because of potential off-target effects of PPARγ agonist and antagonist used in our study, future studies focused on addressing the intracellular mechanisms involved in PPARγ-mediated IL-10 production by TSP-1 are required.

Additionally, *in vivo* data in the context of *K. pneumoniae*-induced pneumonia in mice have shown that the administration of the PPARγ agonist rosiglitazone induced IL-10 production in lung-recruited CD11b^+^Ly6G^int^Ly6C^lo^F4/80^+^ cells, improving host defense and reducing lung inflammation and injury (34). Consistently, during polymicrobial sepsis *in vivo*, the administration of the PPARγ agonist pioglitazone enhanced IL-10 production in serum and lungs, while it reduced blood bacterial burden (48). On the other hand, the administration of the PPARγ antagonist GW9662 or the use of PPARγ^−/−^ mice resulted in an elevated bacterial burden in *Staphylococcus aureus*-caused abscesses, but an improved bacterial clearance when mice were treated with rosiglitazone (49). Based on these data, we speculate that the treatment of Thbs1^−/−^ mice with PPARγ agonist during *P. aeruginosa* infection will enhance lung IL-10 production and host defense while it will downmodulate lung inflammation compared to vehicle-treated Thbs1^−/−^ mice although further studies are needed to corroborate this hypothesis.

Finally, adoptive transfer of *IL-10^+/+^* and *IL-10^−/−^* CD11b^+^Ly6G^+^Ly6C^+^ cells into *Thbs1^−/−^* mice enabled us to examine the physiological role of IL-10-producing myeloid cells in the lungs during acute *P. aeruginosa* infection. While the transfer of *IL-10^+/+^* cells improved lung bacterial clearance, reduced disease severity, increased lung IL-10 production, and decreased lung cytokine levels and lung micro-permeability, these effects were not seen when mice were transferred with *IL-10^−/−^* cells.

These findings suggest that IL-10 production by myeloid cells such as neutrophils and monocytes not only regulates lung inflammation and pro-inflammatory cytokine production but also enhances host defense and diminishes disease severity. Regarding the role of TSP-1 and PAMPs in IL-10 production *in vivo*, we speculate that although TSP-1 may be key for achieving optimal IL-10 production, given that IL-10 can induce its own expression (50), the IL-10 production by transferred cells may promote the production of IL-10 by other cells in the lung of *Thbs1^−/−^* mice.

Whereas IL-10 production by monocytes has been widely reported in several inflammatory models (33, 51), including infectious diseases, the ability of neutrophils to produce IL-10 and being functionally heterogeneous was described much later (18), and even though controversial at first, today it is well-recognized that neutrophils are functionally heterogeneous cells that can acquire either pro-inflammatory signatures characterized by being highly antimicrobial and to cause tissue injury if their function is not regulated; or anti-inflammatory phenotypes able to downmodulate inflammation and to promote injury resolution (17, 29–32). Our multiparametric flow cytometry data are consistent with this notion and suggest heterogeneity even between IL-10-producing neutrophils in the lungs during infection. Interestingly, these analyses also suggest a high heterogeneity between lung Ly6C^+^ monocytes found at baseline when compared with those identified at 24 and 48 hpi.

This anti-inflammatory phenotype of myeloid cells has also been observed in humans, where suppressive granulocytic cells (HLA-DR^neg^Lin^neg^CD33^+^CD11b^+^CD15^+^CD14^neg^) have been identified in both chronic and acute bacterial infections (14), as well as in pulmonary SARS-CoV-2 infection (19), suggesting that in humans, these cells might also play a key role in various other infections.

At present*, P. aeruginosa* is one of the top five causes of bacterial death worldwide due to its widespread presence in healthcare facilities (52). Given the ongoing spread of antimicrobial-resistant *P. aeruginosa*, understanding host factors that enhance the immune response during acute lung infection caused by this bacterium will be a crucial strategy for developing new therapeutic and preventive tools. In this context, our data demonstrate that IL-10, induced by TSP-1 in neutrophils and monocytes, is essential for improving host defense and limiting lung inflammation during infection, establishing a potential pathway for new therapies based on myeloid IL-10-producing cells.

## MATERIALS AND METHODS

### Mice

C57BL/6J wild type (WT) mice, C57BL/6 B6(Cg)-*Il10tm1.1Karp*/J (IL-10-eGFP) mice, C57BL/6 B6.129P2-*Il10tm1Cgn*/J (*IL-10^−/−^*) mice, and C57BL/6 B6.129S2-*Thbs1^tm1Hyn^*/J (*Thbs1^−/−^*) mice were obtained from The Jackson Laboratory (Bar Harbor, ME) and kept in a pathogen-free animal vivarium at the Pontificia Universidad Católica de Chile. All mice were housed in the same institutional vivarium and fed the same chow for at least 4 weeks before experimentation.

### *Pseudomonas aeruginosa* inoculation

*Pseudomonas aeruginosa* (PA14, Bei Resources from the National Institute of Allergy and Infectious Diseases [NIAID]) were grown in LB broth to an optical density of 0.5 (1 × 10^9^ CFU/mL). Fifty microliters of this pre-inoculum was resuspended in 0.95 mL of PBS 1×, reaching a final concentration of 0.5 × 10^7^ CFU/mL (23). Sex-matched 8- to 10-week-old WT and *Thbs1^−/−^* mice were briefly anesthetized with isoflurane in an anesthesia chamber and then intratracheally inoculated with 100 µL of the infection dose (1 × 10^6^ CFU)(23). Mice were euthanized at 24 and 48 h. For experiments involving IL-10^−/−^ mice, WT and IL-10^−/−^ mice were intratracheally inoculated with an adjusted dose of 0.5 × 10^6^ CFU in 100 µL.

### Broncho-alveolar lavage fluid collection

Once mice were euthanized, the trachea was cannulated using a 20-gage catheter. The left lung was clamped to prevent fluid instillation, and then several instillations were performed with PBS-EDTA (0.6 mM), recovering approximately 2 mL of BALF. The cell-free supernatant of BALF was used to measure total proteins (Pierce BCA Protein Assay Kit, Thermo Scientific, 23227) (23).

### *In vivo* lung flow cytometry

Nonperfused left lungs recovered from mice were minced with sterile scissors and incubated in PBS-collagenase (1 mg/mL) for 1 h at 37°C with agitation (250 rpm) (14, 27, 29, 53). Collagenase activity was then inactivated with PBS containing 0.6 mM EDTA. The lung tissue was filtered through a 70 µm cell strainer and mechanically homogenized. The lung homogenate was centrifuged at 1,800 rpm for 10 min at 4°C. Cells were incubated at room temperature with ammonium-chloride-potassium (ACK) lysis buffer (150 mM NH_4_Cl, 10 mM KHCO_3_, and 0.1 mM EDTA) for 5 min, washed with PBS, and centrifuged for 5 min. Cells were stained with Viability Stain 575V (BD Horizon, no 565694), washed twice with PBS, and stained with the following antibodies: CD45 (clone 30-F11, Alexa Fluor 700, BD Pharmingen), Siglec-F (clone E50-2440, APC-Cy7, BD Pharmingen), CD24 (clone M1/69, BUV395, BD OptiBuild), CD64 (clone X54-5/7.1, BV650, BD OptiBuild), Ly6C (clone AL-21, BV421, BD Horizon), Ly6G (clone 1A8, APC, BD Pharmingen), MHC-II (clone M5/114.15.2, Percp-Cy5.5, BD Pharmingen), CD11c (clone HL3, PE-Cy7, BD Pharmingen), and CD11b (clone M1/70, PE, BD Pharmingen). Stained cells were then washed twice with PBS containing 2% FBS and fixed with 2% PFA. Before analysis, CountBright absolute counting beads (Invitrogen, no C36950) were used to quantify cell populations (23). Samples were analyzed using a BD LSR Fortessa flow cytometer available at the Pontificia Universidad Católica de Chile. The gating strategy used to identify populations is described in Fig. S2, and samples were analyzed in Flowjo 10.10.

For the bh-SNE multiparametric analyses (54, 55) on live lung neutrophils and Ly6C^+^ monocytes from *P. aeruginosa*-inoculated IL-10-eGFP mice, gated neutrophils and Ly6C^+^ monocytes (Fig. S2) data were exported as CSV files, which were then used to generate new FSC files for bh-SNE multiparametric analysis using Cyt3.0 (SightOF) software in MatLab R2025b and subsequently visualized and analyzed in Flowjo 10.10.

### Lung cytokine production

The right lung was recovered; the larger and smaller lobes were preserved for lung CFU quantification, while the remaining two lobes were homogenized and used to measure cytokine production. The homogenized lung was resuspended in cytokine lysis buffer (0.5% Triton X-100, 150 mM NaCl, 15 mM Tris, 1 mM CaCl_2_, and 1 mM MgCl_2_ [pH 7.40]), incubated on ice for 30 min, and then centrifuged at 10,000 *g* for 20 min (23). The resulting supernatant was used in ELISA assays to measure the production of IL-10, IL-1β, TNF-α, IL-17, CXCL-1, CXCL-2, G-CSF, and GM-CSF using DuoSet ELISA kits (R&D Systems).

### Bone marrow CD11b^+^Ly6G^+^Ly6C^+^ cell differentiation, adoptive transfer, and *in vitro* stimulation

Mouse CD11b^+^Ly6G^+^Ly6C^+^ cells were differentiated from bone marrow precursors as previously described (16, 33). Briefly, cells were incubated in full RPMI medium supplemented with G-CSF (40 ng/mL) and GM-CSF (40 ng/mL) at 37°C in 5% CO_2_ for 5 days. Cells were recovered and cultured in 24-well plates, 1 × 10^5^ cells per well and stimulated with either vehicle (PBS) or LPS from *Salmonella enterica* (100 ng/mL) (Sigma-Aldrich, n° L7261) at 37°C for 4 h. The supernatant was collected for IL-10 production measurement, while the pellet was used for flow cytometry: CD11b (clone M1/70, Alexa Fluor 700 or PE-Cy7, BD Pharmingen), Ly6C (clone AL-21, BV421 or FITC, BD Horizon), and Ly6G (clone 1A8, APC or PE, BD Pharmingen).

To assess the role of TSP-1 in IL-10 production, bone marrow CD11b^+^Ly6G^+^Ly6C^+^ cells were incubated with recombinant TSP-1 (0, 5, or 15 µg/mL) at 37°C with 5% CO_2_ for 24 h. Then, cells were washed with sterile RPMI media and stimulated with bacterial LPS (100 ng/mL) at 37°C with 5% CO_2_ for 4 h. In parallel, CD11b^+^Ly6G^+^Ly6C^+^ cells were differentiated from WT or Thbs1^−/−^ mice and stimulated with bacterial LPS (100 ng/mL) for 4 h. For each set of experiments, the supernatant was collected, and IL-10 production was measured by ELISA (DuoSet ELISA Mouse IL-10, R&D Systems, DY417).

To assess the role of PPARγ in IL-10 production, bone marrow CD11b^+^Ly6G^+^Ly6C^+^ cells were incubated with recombinant TSP-1 (0 or 15 µg/mL) at 37°C with 5% CO_2_ for 24 h. Then cells were then washed with sterile RPMI media and treated with vehicle (DMSO), the PPARγ agonist GW1929 (100 µM), or the PPARγ antagonist GW9662 (10 µM) for 1 h. All groups were stimulated with bacterial LPS (100 ng/mL) at 37°C with 5% CO_2_ for 4 h. The supernatant was collected for IL-10 measurement via ELISA (DuoSet ELISA Mouse IL-10, R&D Systems, DY417).

For adoptive transfer experiments, 1 × 10^6^ bone marrow *IL-10^+/+^* and *IL-10^−/−^* CD11b^+^Ly6G^+^Ly6C^+^ cells, differentiated from IL-10-eGFP and *IL-10^−/−^* mice, respectively, were intratracheally transferred to *Thbs1^−/−^* mice (16). Twenty-four hours later, mice were inoculated intratracheally with *P. aeruginosa* (1 × 10^6^ CFU). After another 24 h, the mice were euthanized, and the lungs were collected for subsequent analyses. For the identification of transferred CD11b^+^Ly6G^+^Ly6C^+^IL-10^+^ cells in the lungs of *Thbs1^−/−^* mice, cells were gated by flow cytometry and counted using CountBright absolute counting beads (Invitrogen, n°C36950). For final quantification, the mean of IL-10^+^ cells initially identified in *Thbs1^−/−^* + PBS and *Thbs1^−/−^ + IL-10^−/−^* cells was subtracted to those quantified in *Thbs1^+/+^* + PBS (IL-10-eGFP mice) and *Thbs1^−/−^ + IL-10^+/+^* cells to eliminate signal background. FITC-eGFP geometric mean of total gated CD11b^+^Ly6G^+^Ly6C^+^ was quantified in Flowjo 10.10 and Log_2_ normalized as previously described (16).

### Bone marrow neutrophil isolation and culture

Bone marrow from femurs and tibias of WT or IL-10-eGFP mice were flushed with RPMI 1640/10% FBS/EDTA 2 mM media, and erythrocytes were osmotically lysed with a solution of NaCl 0.2% and NaCl 1.6%. Neutrophils were isolated from bone marrow cells using a mouse negative neutrophil isolation kit (MACS Miltenyi Biotec) according to manufacturer instructions. Next, neutrophils were counted, and 5 × 10^5^ cells were plated in 24-well plates in the presence or absence of LPS (100 ng/mL) for 18 h at 37°C 5% CO_2_. After 18 h, neutrophils were counted in a hemocytometer using trypan blue, and cell viability was determined at 18 h.

### Transmission electron microscopy

1 × 10^6^ of differentiated CD11b^+^Ly6G^+^Ly6C^+^ cells were fixed with 2.5% glutaraldehyde in 0.1 M sodium cacodylate buffer (pH 7.2). Fixed cells were prepared and examined using a Philips Tecnai 12 (Biotwin) transmission electron microscope at the Advanced Microscopy Unit, Pontificia Universidad Católica de Chile.

### Statistical analyses

For simple comparison, a two-tailed *t*-test analysis was performed. For multiple comparisons, a one-way analysis of variance (ANOVA) followed by a Holm Sidak post-hoc test or a two-way ANOVA with Sidak post-hoc test was used, depending on the data distribution. A *P* value < 0.05 was considered statistically significant. All comparisons were conducted using Prism software v10.2.2 (GraphPad Software).

## Data Availability

The data presented in this study are available from the corresponding author upon request.

## SUPPLEMENTAL MATERIAL

Supplemental material iai.00614-25-s0001.pdf Fig. S1 to S8; Table S1.

ASM does not own the copyrights to Supplemental Material that may be linked to, or accessed through, an article. The authors have granted ASM a non-exclusive, world-wide license to publish the Supplemental Material files. Please contact the corresponding author directly for reuse.
