# Supplementary material for: Myeloid-derived interleukin-10 induced by thrombospondin-1 mediates host defense and regulates inflammation during acute bacterial lung infection
Source: Infect Immun. 2026 Feb 24;94(4):e00614-25. doi: 10.1128/iai.00614-25 (PMC13081729; doi:10.1128/iai.00614-25)
Supplement: Supplemental material — Fig. S1 to S8; Table S1. [file iai.00614-25-s0001.pdf]

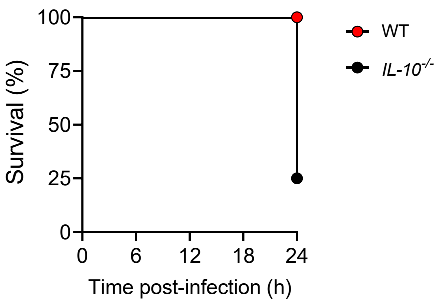

Supplementary figure 1. Bone marrow neutrophil viability in culture over time. Survival of WT and *IL-10$^{-/-}$* mice inoculated with 1x10^{6} CFU of *P. aeruginosa* PA14. WT (n=4) and *IL-10$^{-/-}$* (n=4) mice were intratracheally inoculated with 1x10^{6} CFU of *P. aeruginosa* and survival was measured during the first 24 hours post infection.

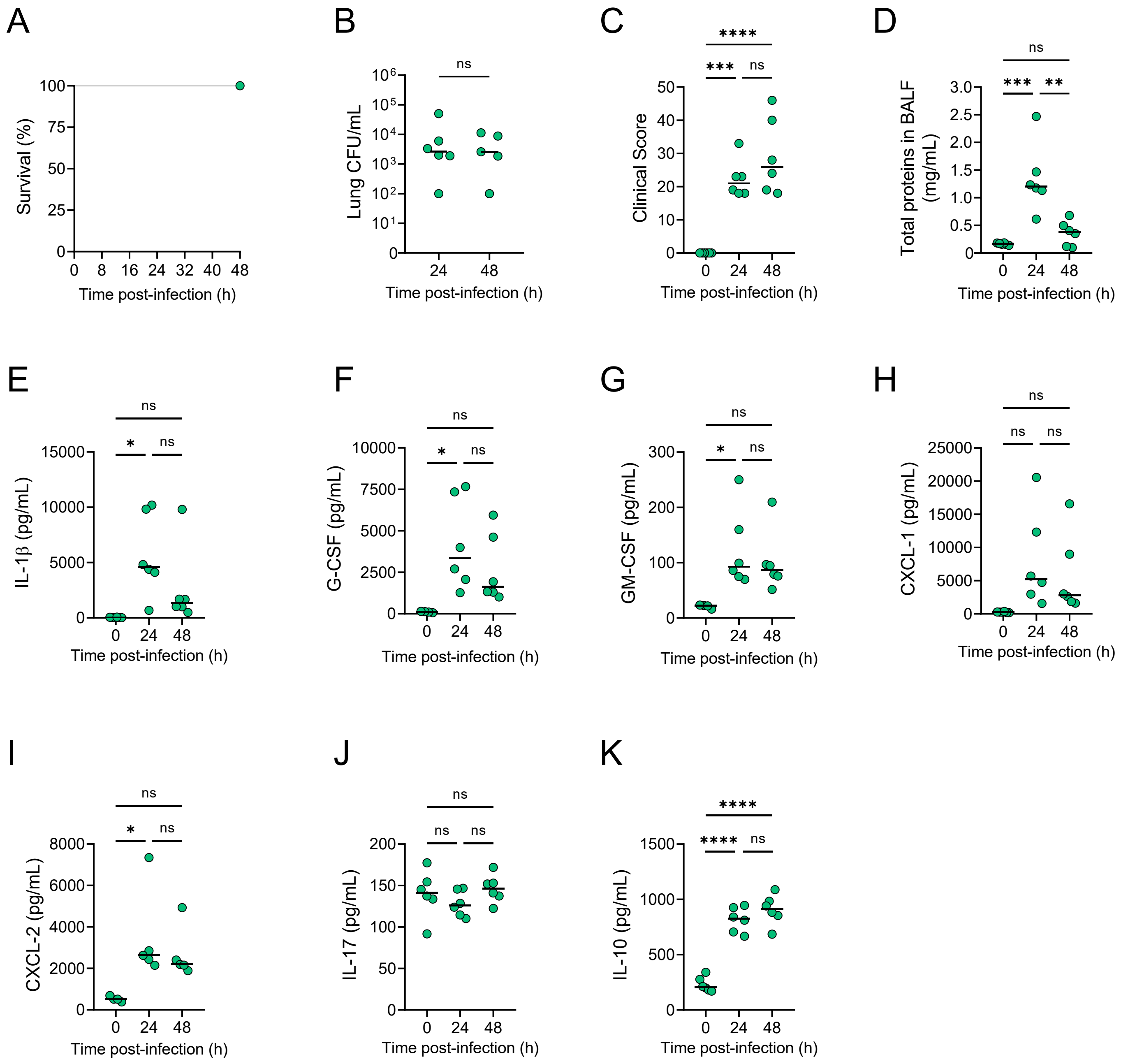

Supplementary figure 2. Host defense and inflammatory response of IL-10-eGFP mice against *Pseudomonas aeruginosa* infection. IL-10-eGFP mice were intratracheally inoculated with $1x10^6$ CFU. (A) Survival of IL-10-eGFP mice during the first 48 hours post-infection, (B) lung bacterial burden (CFU/mL), (C) disease severity (clinical score) and (D) total BALF protein content was measured at 0, 24 and 48 hours post-infection. Lung (E) IL-1β, (F) G-CSF, (G) GM-CSF, (H) CXCL-1, (I) CXCL-2, (J) IL-17 and (K) IL-10 were evaluated in lung homogenates at 24 and 48 hpi by ELISA. Analysis of variance (ANOVA) followed by a Holm Sidak post-hoc test for multiple comparisons over time (ns $p > 0.05$, * $p < 0.05$, ** $p < 0.01$, *** $p < 0.001$). Each data point represents an individual mouse, combined from two independent experiments (n=6 for each time point). Lines indicate the median.

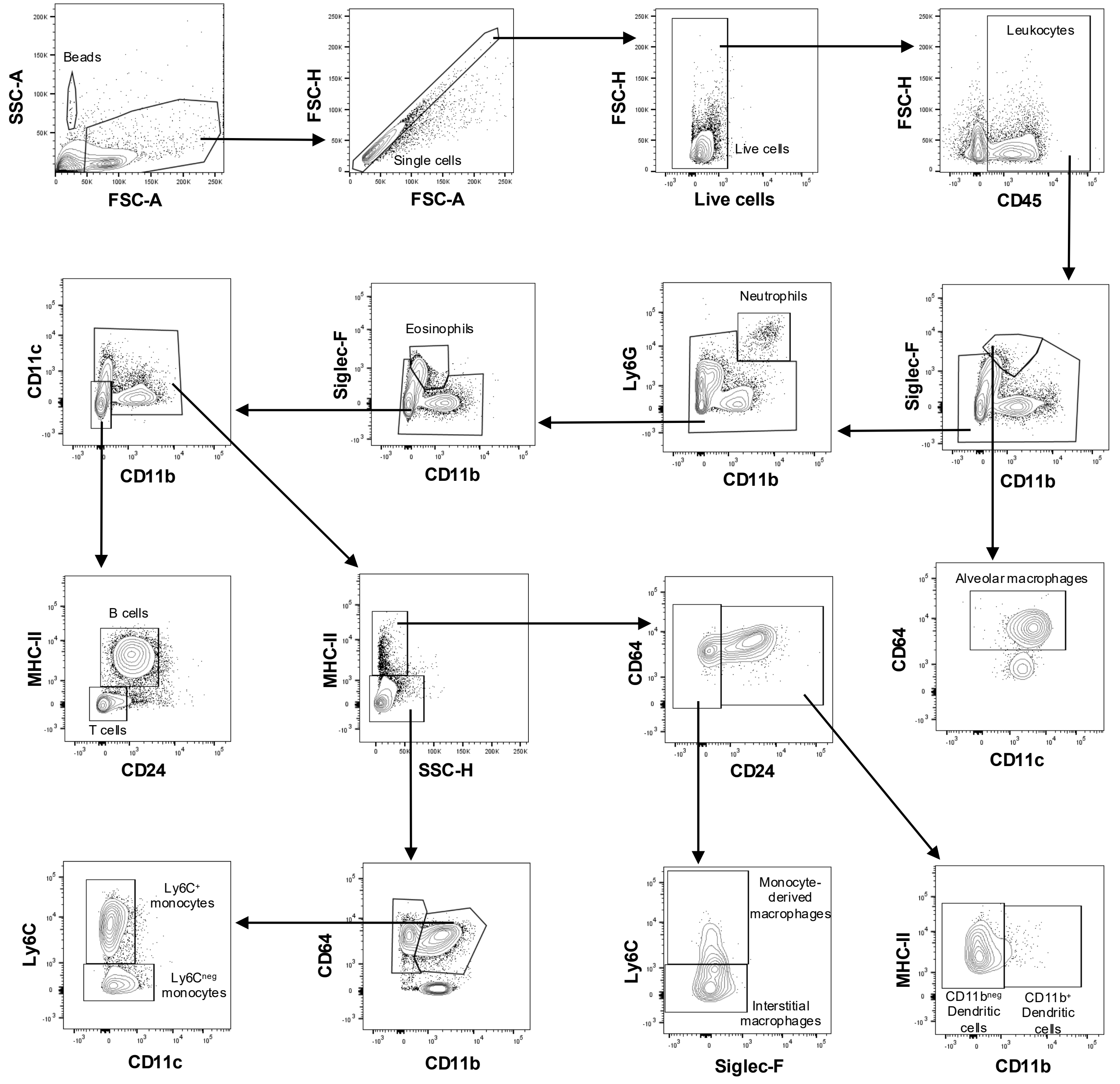

Supplementary figure 3. Gating strategy for immune cell identification in lungs. Live cells were identified with a Viability Stain 575V (BD Horizon, n°565694) and leukocytes ($CD45^+$ cells) were gated based on the expression of CD45 (clone 30-F11, Alexa Fluor 700, BD Pharmingen). To specifically identify alveolar macrophages, neutrophils, eosinophils, B and T cells, $CD11b^+$ and $CD11b^{neg}$ dendritic cells, $Ly6C^+$ and $Ly6C^{neg}$ monocytes, interstitial macrophages and monocyte-derived macrophages the following antibodies were used: Siglec-F (clone E50-2440, APC-Cy7, BD Pharmingen), CD24 (clone M1/69, BUV395, BD OptiBuild), CD64 (clone X54-5/7.1, BV650, BD OptiBuild), Ly6C (clone AL-21, BV421, BD Horizon), Ly6G (clone 1A8, APC, BD Pharmingen), MHC-II (clone M5/114.15.2, Percp-Cy 5.5, BD Pharmingen), CD11c (clone HL3, PE-Cy7, BD Pharmingen), CD11b (clone M1/70, PE, BD Pharmingen). Cells/lung were calculated using CountBright absolute counting beads (Life Technologies).

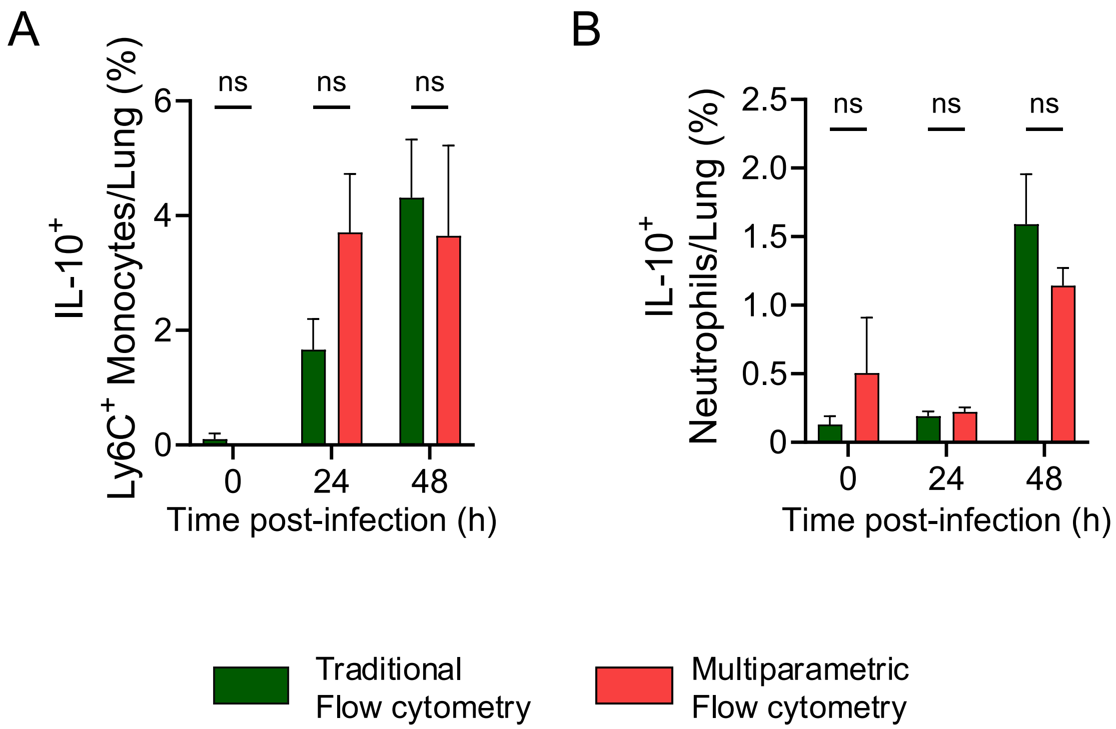

Supplementary figure 4. Evaluation of IL-10 production by neutrophils and monocytes in lung by traditional and multiparametric flow cytometry. Percentage of lung IL-10^{+} (A) neutrophils and (B) Ly6C^{+} monocytes evaluated in IL-10-eGFP mice by traditional and multiparametric flow cytometry at 0, 24 and 48 hpi. A two-tailed t-test was used for single comparisons (ns p>0.05). Each data point represents one individual mouse. mean + SE.

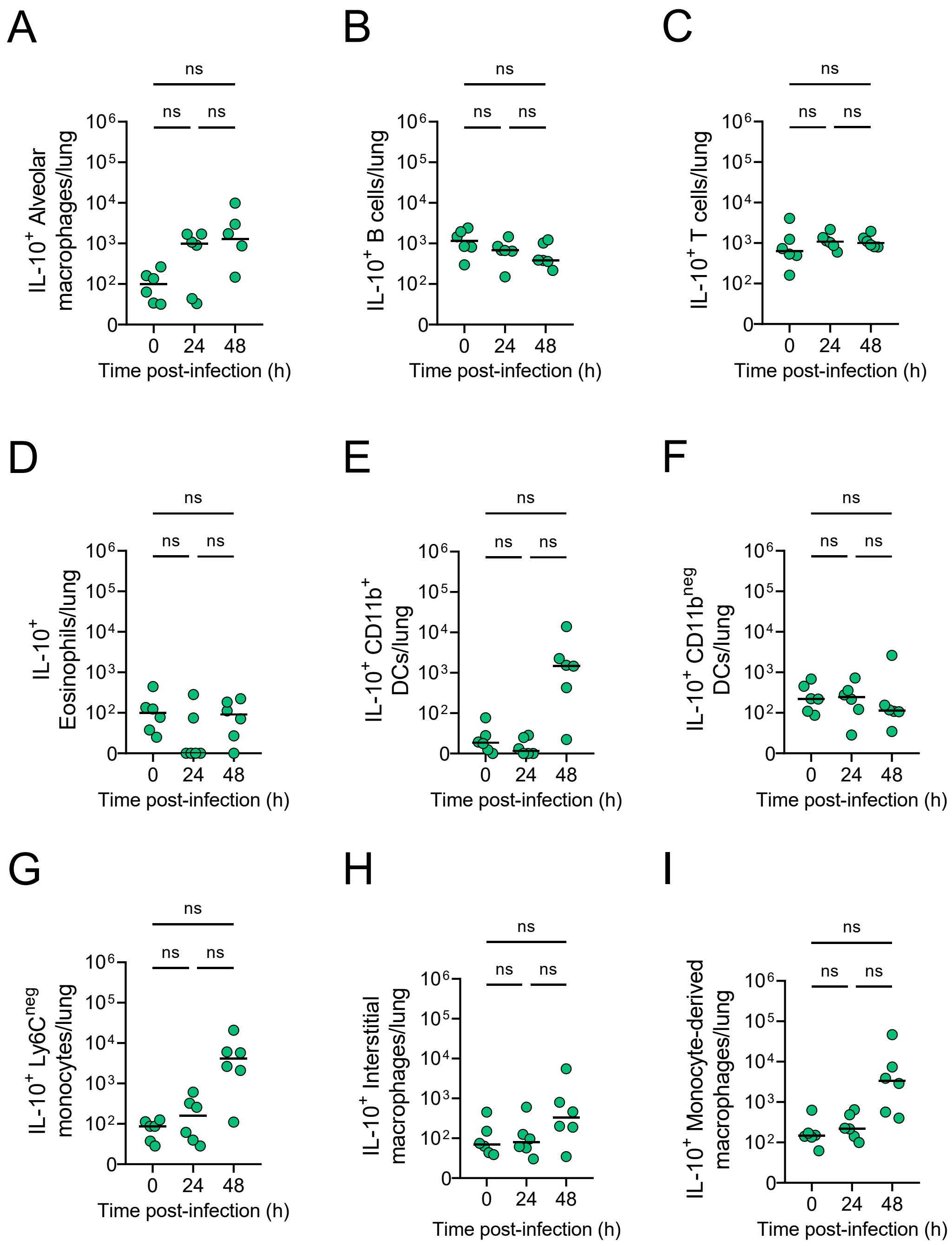

Supplementary figure 5. IL-10 production in the lungs by other immune cells during *P. aeruginosa* infection. IL-10 production by (A) alveolar macrophages, (B) B cells, (C) T cells, (D) eosinophils, (E) $CD11b^{+}$ and (F) $CD11b^{neg}$ dendritic cells, (G) $Ly6C^{neg}$ monocytes, (H) interstitial macrophages and (I) monocyte-derived macrophages was evaluated in IL-10-eGFP mice by flow cytometry. A one-way analysis of variance (ANOVA) test followed by a Holm Sidak post-hoc for multiple comparisons was performed for multiple comparisons over time (ns $p > 0.05$). Each data point represents an individual mouse (n=6 for each group), combined from two independent experiments. Lines indicate the median.

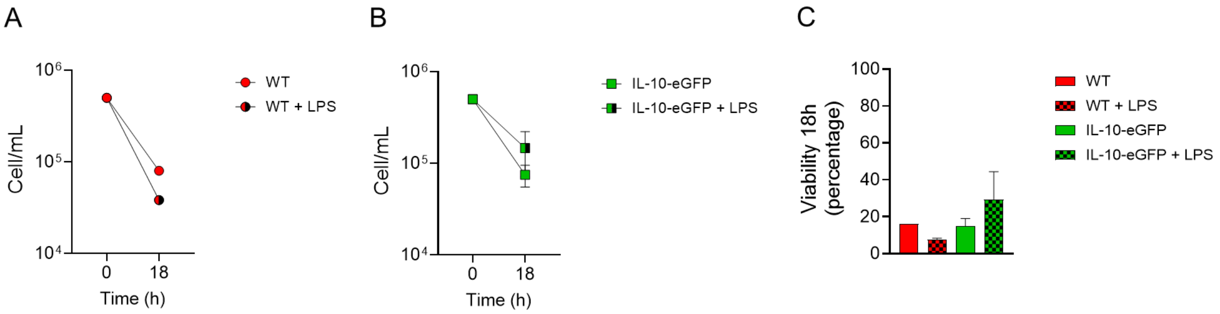

Supplementary figure 6. Bone marrow neutrophil viability in culture over time. Cell count of (A) WT- and (B) IL-10-eGFP-derived bone marrow neutrophils stimulated with LPS or vehicle at 0 and 18 hours. $5x10^5$ neutrophils were incubated in 24 well-plates (0h) and stimulated with vehicle or LPS (100 ng/mL) for 18h at 37ºC 5% CO_2. At 0h and after 18h of stimulation, neutrophils were counted in a hemocytometer using trypan blue. (C) Cell viability of WT and IL-10-eGFP bone marrow neutrophils at 18h was determined considering that 100% viability the number of cells initially seeded at time 0h ($5x10^5$ cells/well).

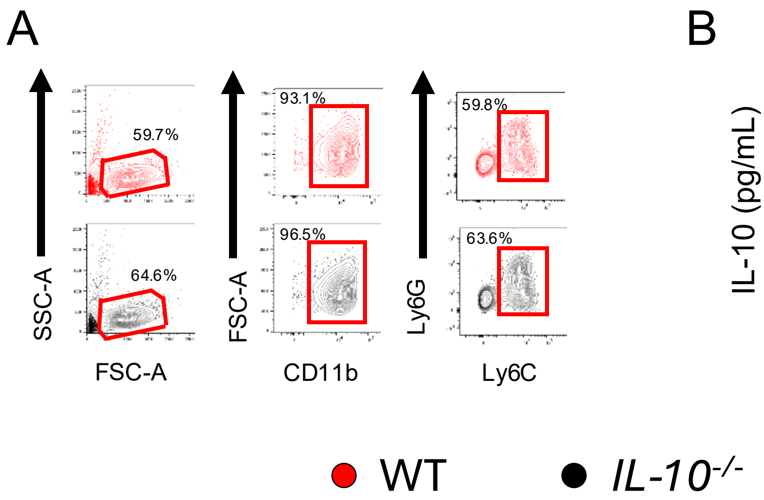

Supplementary figure 7. IL-10 production by CD11b⁺Ly6G⁺Ly6C⁺ cells in response to *P. aeruginosa*. CD11b⁺Ly6G⁺Ly6C⁺ cells from WT and *IL-10*$^{-/-}$ mice were stimulated with vehicle (PBS) and bacterial LPS (100 ng/mL) for 4 hours. Differentiated cells were phenotypically characterized by (A) flow cytometry and (B) IL-10 production in the supernatant was evaluated by ELISA. Two-tailed t-test was used to evaluate IL-10 production between stimulus (ns $p > 0.05$, * $p < 0.05$, ** $p < 0.01$, *** $p < 0.001$). Each data point represents a technical replicate, combined from three independent experiments. Lines indicate the median.

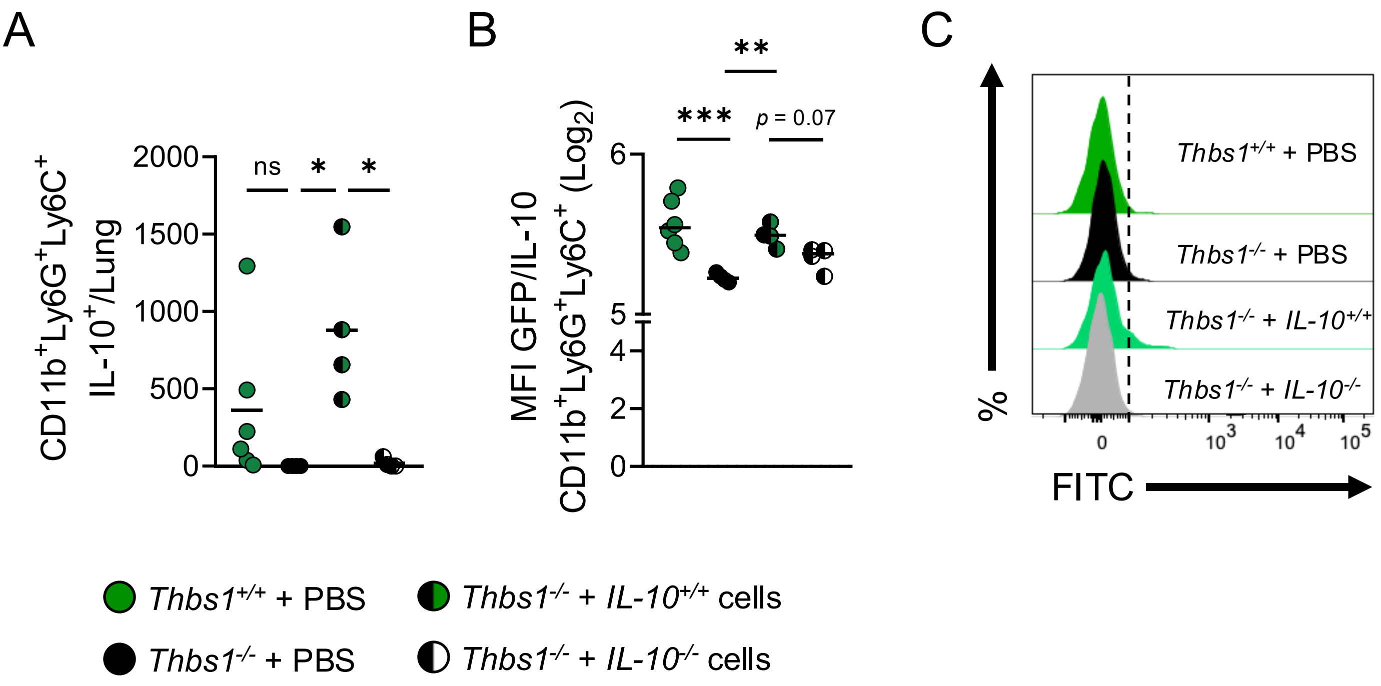

Supplementary figure 8. IL-10 production by CD11b$^+$Ly6G$^+$Ly6C$^+$ cells in lungs of *Thbs1$^{-/-}$* transferred mice. *IL-10$^{+/+}$* and *IL-10$^{-/-}$* CD11b$^+$Ly6G$^+$Ly6C$^+$ cells were differentiated and intratracheally transferred to *Thbs1$^{-/-}$* mice (non-transferred IL-10-eGFP mice (*Thbs1$^{+/+}$*) were used as controls). 24 hours after transfer, mice were intratracheally inoculated with 1x10^6 CFU of PA14. (A) Count of transferred CD11b$^+$Ly6G$^+$Ly6C$^+$IL-10$^+$ cells found in lung based on eGFP expression and (B) IL-10 (FITC) Median fluorescence intensity (MFI) by CD11b$^+$Ly6G$^+$Ly6C$^+$ cells in the lungs of transferred and control mice and (C) representative histograms of eGFP/FITC fluorescence of lung CD11b$^+$Ly6G$^+$Ly6C$^+$ cells. One-way analysis of variance (ANOVA) test followed by Holm Sidak post-hoc for multiple comparisons was performed for multiple comparisons by group (ns p>0.05, * $p < 0.05$, ** $p < 0.01$, *** $p < 0.001$). Each data point represents an individual mouse.

Supplementary table 1. Clinical parameters considered for evaluating disease severity in mice.

Parameters	Score
**I. General conditions**	
Coat	
Shiny	0
Dull and opaque	2
Messy and unkempt	4
Eyes	
Clear and bright	0
Dirty, with eye discharge	3
Semi-closed	5
Posture	
Normal	0
Hunched	10
Highly hunched	20
**II. Mobility**	
Spontaneous mobility (normal and social behavior)	0
Spontaneous but reduced mobility	1
The mouse moves only after being stimulated	5
The animal is isolated, lethargic, and has motor coordination problems	10
**III. Respiration**	
Normal	0
Slightly altered breathing rate	1
Rapid breathing (tachypnea)	10
Abdominal breathing or gasping (agonal breathing)	20
**IV. Body weight**	
Does not change	0
Weight loss in percentage, where the score is equal to the percentage	1 - 20
Weight loss greater than 20%	20
